# A Health Economic Analysis for Oral Poliovirus Vaccine to Prevent COVID‐19 in the United States

**DOI:** 10.1111/risa.13614

**Published:** 2020-10-20

**Authors:** Kimberly M. Thompson, Dominika A. Kalkowska, Kamran Badizadegan

**Affiliations:** ^1^ Kid Risk, Inc. Orlando Florida 32819 USA

**Keywords:** COVID‐19, health economics, oral poliovirus vaccine, polio

## Abstract

COVID‐19 led to a recent high‐profile proposal to reintroduce oral poliovirus vaccine (OPV) in the United States (U.S.), initially in clinical trials, but potentially for widespread and repeated use. We explore logistical challenges related to U.S. OPV administration in 2020, review the literature related to nonspecific effects of OPV to induce innate immunity, and model the health and economic implications of the proposal. The costs of reintroducing a single OPV dose to 331 million Americans would exceed $4.4 billion. Giving a dose of bivalent OPV to the entire U.S. population would lead to an expected 40 identifiable cases of vaccine‐associated paralytic polio, with young Americans at the highest risk. Reintroducing any OPV use in the U.S. poses a risk of restarting transmission of OPV‐related viruses and could lead to new infections in immunocompromised individuals with B‐cell related primary immunodeficiencies that could lead to later cases of paralysis. Due to the lack of a currently licensed OPV in the U.S., the decision to administer OPV to Americans for nonspecific immunological effects would require purchasing limited global OPV supplies that could impact polio eradication efforts. Health economic modeling suggests no role for reintroducing OPV into the U.S. with respect to responding to COVID‐19. Countries that currently use OPV experience fundamentally different risks, costs, and benefits than the U.S. Successful global polio eradication will depend on sufficient OPV supplies, achieving and maintaining high OPV coverage in OPV‐using countries, and effective global OPV cessation and containment in all countries, including the U.S.

## 1 INTRODUCTION

Over the last 20 years, an extensive body of integrated poliovirus transmission dynamic modeling and health economic policy analyses explored the benefits, risks, and costs of various poliovirus vaccines (Thompson & Kalkowska, 2020b, 2020c). The prior modeling considers the differences in the immunity induced by the different formulations of oral poliovirus vaccine (OPV) and by inactivated poliovirus vaccine (IPV) (Thompson & Duintjer Tebbens, 2012). OPV, which uses attenuated live poliovirus strains, comes in a liquid form that allows easy delivery of two drops by mouth. IPV delivery requires an injection by trained health workers, although recent research (Edens, Dybdahl‐Sissoko, Weldon, Oberste, & Prausnitz, 2015; Muller et al., 2017) explores the potential to deliver IPV using a vaccine patch (Badizadegan, Goodson, Rota, & Thompson, 2020). In the United States (U.S.), historical and ongoing polio control and elimination efforts have yielded substantial health and economic benefits (Thompson & Duintjer Tebbens, 2006).

Prior to the introduction of poliovirus vaccines, the transmission of wild polioviruses led to devastating outbreaks that caused paralysis in approximately 1 in 200 to 1 in 2,000 infections of fully susceptible individuals depending on the poliovirus serotype (serotype 1 > 3 > 2), with an overall rate of approximately 1 case of paralysis per 150 poliovirus infections (Nathanson & Kew, 2010). In the mid‐1900s, hospitals in the U.S. maintained “polio wards” that operated iron lungs, and the fear caused by polio outbreaks motivated Americans to send dimes to support the development of polio vaccines (Oshinsky, 2005). The terror of polio, now largely unappreciated by all but some of the oldest Americans, led parents to restrict the activities of their children, because polio paralyzed only some individuals, while most infections occurred asymptomatically (Oshinsky, 2005). The introduction of IPV in 1954 followed by a shift to OPV in the early 1960s dramatically reduced wild poliovirus (WPV) transmission and paralysis cases in the U.S. (Thompson & Duintjer Tebbens, 2006). Poliovirus vaccine use in the U.S. (and other countries that achieved high OPV immunization coverage) eliminated national indigenous wild poliovirus transmission by infecting susceptible individuals with less transmissible and less neurovirulent live poliovirus strains.

In spite of its effectiveness as a vaccine against polio, the risks of OPV use include vaccine‐associated paralytic polio (VAPP), which occurs in approximately 1 per million infections in fully susceptible individuals (i.e., those never exposed to a live poliovirus or vaccinated against polio). VAPP poses a risk directly to OPV vaccine recipients, and also indirectly to those who come in contact with OPV‐infected individuals for as long as the vaccine‐derived viruses continue to transmit secondarily (Platt, Estivariz, & Sutter, 2014; Duintjer Tebbens et al., 2006). In addition, individuals with B‐cell primary immunodeficiencies (PIDs) can experience prolonged or chronic infection with OPV (i.e., immunodeficiency‐associated long‐term vaccine‐derived poliovirus or iVDPV), which can lead to these individuals becoming paralyzed with VAPP (called iVAPP due to the association with PID) (Duintjer Tebbens, Pallansch, & Thompson 2015; Kalkowska, Pallansch, & Thompson, 2019; Duintjer Tebbens et al., 2006). Finally, in populations with relatively low OPV coverage, OPV‐related viruses can mutate and lose their attenuating mutations as they transmit through the population. As these vaccine‐derived polioviruses (VDPVs) evolve, they can become circulating VDPVs (i.e., cVDPVs), which behave like wild polioviruses with respect to transmission potential and neurovirulence (Duintjer Tebbens et al., 2013; Kew et al., 2002; Duintjer Tebbens et al., 2006). In the absence of polio cases caused by wild polioviruses, starting in 1994, the only reported cases of poliomyelitis in the U.S. were due to VAPP (i.e., no wild poliovirus cases) (Thompson, 2015). The U.S. did not experience any cases from VDPVs while using OPV up until 2000 due to very high immunization coverage and conditions that led to relatively low transmission potential of live polioviruses.

Although the U.S. eliminated indigenous wild poliovirus transmission by 1980 (Thompson & Duintjer Tebbens, 2006), poliovirus vaccination continues with high coverage in the U.S. due to the risk of importations. Specifically, despite ongoing efforts of the Global Polio Eradication Initiative (GPEI), serotype 1 wild poliovirus (WPV1) remains endemic in parts of Pakistan and Afghanistan, and recent modeling anticipates its continued transmission for the foreseeable future (Kalkowska, Wassilak, Cochi, Pallansch, & Thompson, 2020). In the U.S. and most high‐income countries, national immunization programs currently recommend IPV as the only polio vaccine in their routine immunization schedules (i.e., no OPV) (Kalkowska et al., 2020). As an inactivated vaccine, IPV does not cause infection or spread secondarily. In the U.S., recommendations made by the Advisory Committee on Immunization Practices (ACIP) determine the U.S. vaccine schedule and eligibility for purchase by the Vaccines for Children Program, which guarantees full access to all childhood vaccines. The ACIP evaluates the safety of vaccines as well as their risks and benefits as part of its recommendations, which change as conditions and vaccine products evolve over time. ACIP recommendations also determine the list of vaccines included for no‐fault compensation for injuries associated with recommended vaccines under the Vaccine Injury Compensation Program (VICP) (Thompson, Orenstein, & Hinman, 2020). Early experience with poliovirus vaccines in the U.S. led to some of the foundational case law related to vaccine injury compensation (Offit, 2005), and compensation for VAPP under VICP helped to keep OPV manufacturers in the U.S. market until 2000.

The use of IPV only for polio immunization for the last 20 years in the U.S. provided sufficient protection to prevent whatever importations of live polioviruses occurred from restarting chains of live poliovirus transmission (Thompson et al., 2012; Thompson, Kalkowska, & Duintjer Tebbens, 2015) and the risks of restarting transmission in the U.S. remain very low. However, Israel, which adopted an IPV‐only schedule in 2005, faced different importation and transmission risks and detected widespread asymptomatic transmission of WPV1 imported from Syria in 2013 (Anis et al., 2013). After efforts to stop the WPV1 transmission using IPV only failed, Israel reintroduced OPV using a sequential IPV/OPV schedule, which ended the WPV1 transmission (Kalkowska et al., 2015).

In 1982, the ACIP recommended that “Patients with immune‐deficiency diseases, such as combined immunodeficiency, hypogammaglobulinemia and agammaglobulinemia, should not be given OPV because of their substantially increased risk of vaccine‐associated disease” (Centers for Disease Control and Prevention, 1982). By the mid‐1990s, the ACIP concluded that the risks of VAPP exceeded the benefits of OPV use in the U.S. given the availability of IPV, and consequently the ACIP changed its recommendations to exclusively recommend IPV use (Centers for Disease Control and Prevention, 2000). Thus, despite a long safety record of OPV use, the elimination of all indigenous WPV transmission in the U.S. shifted the national risk tolerance for VAPP given the availability of IPV as an option. This decision did not come lightly, because IPV does not induce the same type of immunological protection as OPV and it costs much more to produce and administer (Centers for Disease Control and Prevention, 2000; Miller, Sutter, Strebel, & Hadler, 1996). Since 2000, no vaccine manufacturer has offered a licensed OPV for the U.S. market. Continued OPV use in global markets supported the ACIP recommendation in 2000 to allow for potential OPV use for mass vaccination in the event of a polio outbreak in the U.S. (Centers for Disease Control and Prevention, 2000). However, at the time of a 2006 decision analysis related to outbreak response in the U.S. that recommended stockpiling OPV (Jenkins & Modlin, 2006), no US OPV stockpile existed and no vaccine manufacturers were willing to relicense OPV in the U.S. These conditions remain unchanged.

In general, due to their use of IPV only for many years (e.g., 20 years in the U.S.), high‐income countries that provide lifesaving therapies to individuals with PIDs face low and decreasing risks of iVAPP cases or iVDPVs (Kalkowska et al., 2019). Any decision to use OPV in the U.S. (or other high‐income countries that exclusively use IPV) should follow careful consideration of the risks of OPV use, including restarting live poliovirus infections in immunocompromised individuals and the potential for transmission, as well as the expected costs and benefits.

Recently, the coronavirus disease 2019 (COVID‐19) pandemic led to a proposal to reintroduce OPV first in clinical trials (Study NCT04540185, scheduled to begin on November 1, 2020) and potentially on a mass scale in the U.S. (Chumakov, Benn, Aaby, Kottilil, & Gallo, 2020). The health and economic implications of such use remain uncharacterized. Given the high visibility of the proposal, which included a cost estimate of on the order of “$13 million to vaccinate the entire U.S. population” (Sullivan, 2020) that “looks like a free” option (Gallo & Arbess, 2020), and the possibility of “repeat vaccination” (Gallo & Arbess, 2020) that could include giving it “again and again and again” (Cohn, 2020), we characterize the health and economic impacts of this proposal.

## 2 METHODS

We explore the current status and availability of OPV for potential use in the U.S. market. Using cost information about poliovirus vaccines (Thompson & Kalkowska, 2020a) and knowledge about the VICP (Thompson et al., 2020), we characterize the expected costs per dose of the proposal (Chumakov et al., 2020; Sullivan, 2020) to purchase and administer OPV to the entire estimated U.S. population of 331 million people using 2019 U.S. dollars ($). We systematically review the literature related to the potential nonspecific effects of OPV use related to COVID‐19 to characterize the potential benefits of OPV use in the U.S. population in 2020 for purposes of health economic modeling related to COVID‐19. Using models previously developed for poliovirus transmission in the U.S. population (Kalkowska et al., 2020; Duintjer Tebbens et al., 2013; Thompson & Duintjer Tebbens, 2006; Thompson et al., 2015; Thompson et al., 2012), we estimate the expected health outcomes associated with administering OPV to the entire U.S. population, which currently includes 20 birth cohorts with no or very limited exposure to any live polioviruses. We estimate the number of VAPP cases likely to occur and the expected new iVDPV infections created if these individuals become infected with OPV.

## 3 RESULTS

### 3.1 Availability of licensed OPV for the United States

Consistent with ACIP recommendations (Centers for Disease Control and Prevention, 2000), no vaccine manufacturers currently offer a licensed OPV for use in the U.S. Consequently, any potentially interested OPV manufacturer would need to perform all of the necessary regulatory and licensing steps prior to offering OPV in the U.S. This would likely require 18–24 months for an existing OPV manufacturer, of which only a few currently continue to provide any OPV to the global market. We estimate that the cost of the regulatory and licensing steps alone would most likely exceed the $13 million suggested in popular media coverage as the cost for administering OPV to all Americans (Sullivan, 2020) by a factor of at least three, and probably more, if any OPV manufacturer would even consider reentry of OPV into the U.S. market.

If the U.S. could procure sufficient OPV supply for off‐label use for all Americans, this would come out of a limited global supply produced based on orders from OPV‐using countries or the GPEI to support polio eradication. Due to the planned coordinated cessation of all OPV after successful WPV eradication (Thompson & Duintjer Tebbens, 2008; World Health Assembly, 2008), and completed phase out of all serotype 2‐containing OPV in 2016 (except for emergency use to respond to a serotype 2 outbreak) (Farrell et al., 2017), OPV‐using countries currently use bivalent OPV (bOPV) containing serotypes 1 and 3 for routine immunization. Consequently, currently bOPV remains the licensed OPV product with the largest global supply. Due to concerns about conducting OPV immunization campaigns that could inadvertently increase contacts and thus increase SARS‐CoV‐2 transmission, the GPEI cancelled some polio immunization activities in 2020, and prioritized using GPEI‐funded staff to support national COVID‐19 response activities. This action may imply some unexpected excess supply of bOPV, in addition to excess supply caused by unplanned cancellation of immunization activities in 2019 (UNICEF, 2019), although countries are reporting bOPV stock‐outs (World Health Organization Global Polio Eradication Initiative, 2020). Theoretically, the U.S. might also seek to use monovalent OPV (mOPV) from global stockpiles created to support outbreak response (Thompson & Duintjer Tebbens, 2008), although these stockpiles exist to manage polio outbreaks and remain tightly controlled. In general, any unplanned use of large quantities of vaccines creates challenges for the supply chain, and large orders by high‐income countries could substantially distort access for relatively lower income countries.

### 3.2 Cost Estimates

Pursuing the proposal of giving OPV to the entire U.S. population synchronously (Chumakov et al., 2020) (assuming a positive clinical trial result) and based on the implicit hypothetical assumption of unlimited supply of a licensed OPV supply that the U.S. could purchase, we assume the need to purchase 331 million doses for a single round of vaccination. Payments for the VICP would cost $0.75 per dose (Thompson et al., 2020), which leads to a cost of $248 million for the insurance alone to compensate the identifiable individuals who will present with VAPP. We estimate a minimum average price of $8.75 per dose based on a recent prior estimate for high‐income countries (Thompson & Kalkowska, 2020a). This would imply over $2.9 billion for the OPV itself. This estimate may prove low since the cost per OPV dose prior to its discontinuation in the U.S. exceeded approximately $4 and $17 per dose for the public and private sectors, respectively (prices reported elsewhere (Miller et al., 1996; Thompson & Duintjer Tebbens, 2006) converted to US$2019 and also see, Dreazen, 1999). Assuming a 50% public–private mix (Miller et al., 1996; Thompson & Duintjer Tebbens, 2006) thus implies over $10 per dose on average based on the historical U.S. prices. Finally, we estimate administration costs of at least $4 per dose, which implies $1.3 billion to administer the OPV. This sums to an estimated cost of over $4.4 billion for a single round of OPV vaccination for the entire U.S. population, which exceeds the $13 million suggested in popular media coverage (Sullivan, 2020) by a factor of 342. These costs would repeat for each OPV dose given to the U.S. population to obtain temporary effects. The authors of the perspective (Chumakov et al., 2020) assumed that the U.S. would purchase and administer OPV at a cost less than $0.15 per dose (Cohn, 2020; Sullivan, 2020) (i.e., at the level of the OPV vaccine purchase price for low‐income countries), without consideration of actual U.S. vaccine prices or delivery and insurance costs. Tiered vaccine pricing supports much lower OPV prices in lower income countries, but we do not expect that any OPV manufacturers would agree to license and sell OPV in the U.S. at prices substantially below those in effect prior to the time that the U.S. discontinued OPV use in 2000. As noted above, our vaccine price estimate more likely represents a lower bound.

Recognizing the contraindications of giving OPV to all Americans (Centers for Disease Control and Prevention, 1982, 2000), we implicitly assume that doses purchased for these individuals would offset any vaccine wastage. We also ignore the costs associated with individuals taking time off from productive activities and/or traveling to get OPV, any additional costs to the health system for personal protective equipment and training, and any isolation costs incurred by individuals with OPV contraindications (notably those with PIDs) to minimize their risks of exposure to OPV‐related viruses excreted by vaccine recipients that transmit secondarily.

### 3.3 Health Consequences of Reintroducing OPV in the United States

Using existing poliovirus transmission models applied to the U.S. (Kalkowska et al., 2020; Duintjer Tebbens et al., 2013; Thompson & Duintjer Tebbens, 2006; Thompson et al., 2015; Thompson et al., 2012), Table I shows estimates of the percent of the total U.S. population that remains fully susceptible by serotype. Consistent with serological evidence from a 2009–2010 national survey (Wallace, Curns, Weldon, & Oberste, 2016), Table I shows differences by serotype (3 > 1 > 2) that reflect the different polio vaccine take rates and transmission potential for the serotypes. Our estimates of fully susceptible individuals fall below the estimates implied by simply assuming the 2009–2010 serological measurements (Wallace et al., 2016). We attribute our higher estimates of immunity (and thus lower estimates of fully susceptible individuals) to more birth cohorts receiving IPV with higher take rates and high coverage since the collection of the data over a decade ago, and to levels of detection applied in the interpretation of serological testing results.

**Table I:** Estimates of vaccine‐associated paralytic polio (VAPP) cases associated with delivering a single dose of OPV to the entire U.S. population in 2020

Vaccine, Serotype	Percent of U.S. Population Fully Susceptible (%)	Take Rate	VAPP Rate Per Fully Susceptible Individual	Expected VAPP Cases
bOPV, Serotype 1	5.4	0.80	7.40E‐08	1
bOPV, Serotype 3	11.3	0.80	1.30E‐06	39
Total for bOPV				40
				
mOPV, Serotype 1	5.4	0.90	7.40E‐08	1
mOPV, Serotype 2	4.0	0.95	6.20E‐07	8
mOPV, Serotype 3	11.3	0.85	1.30E‐06	41

Table I also shows our assumptions for the take rates for each serotype for bOPV or mOPV, and our assumed VAPP rate per fully susceptible individual by serotype. We assume that only fully susceptible individuals could become paralyzed because any prior exposure to live polioviruses (including OPV) or immunization with IPV provides lifelong protection from paralysis (Kalkowska et al., 2020). The number of VAPP cases depend on the serotype(s) of OPV included in the vaccine dose. We estimate that giving bOPV to 331 million Americans in mid‐2020 would lead to an expected 40 VAPP cases, which in turn would result in VICP claims that would require compensation. For context, Table I also includes estimates of VAPP cases in the event of mOPV use in 2020 by serotype. The individuals paralyzed by OPV and OPV‐related viruses would occur predominantly in young Americans and be identifiable (Schudel, 2018), which would contrast with any statistical health benefits associated with OPV use for SARS‐CoV‐2 infection prevention. Due to high coverage with IPV, the prior immunity induced will prevent paralysis in most Americans under 21 years old, but we still expect that nearly all young Americans could participate widely in asymptomatic transmission, with the transmission dynamics dependent on actual coverage. Failing to completely isolate and exclude contraindicated individuals, including those with currently undiagnosed PIDs, could also seed new iVDPV excreters who could remain chronically and asymptomatically infected until they clear the infection or develop iVAPP. Based on our prior modeling of iVDPVs (Kalkowska et al., 2019) and available survey data (Modell, Orange, Quinn, & Modell, 2018), we estimate a prevalence of over 10,000 Americans with B‐cell related PIDs who could potentially become iVDPVs if infected with OPV or OPV‐related viruses. In September 2020, a clinical trial proposing to use OPV in the U.S. (NCT04540185) provided explicit exclusion of immunocompromised individuals, but did not exclude individuals who might personally or professionally interact with immunocompromised individuals.

### 3.4 Nonspecific Effects of OPV

We searched PubMed® and systematic reviews commissioned by the World Health Organization (WHO) (Higgins et al., 2016; Kandasamy et al., 2016) for studies related to OPV and its potential non‐specific effects. We identified two randomized clinical trials (Lund et al., 2015; Upfill‐Brown et al., 2017) and numerous other studies.

Lund et al. (2015) describe a randomized nonblind clinical trial comparing the effects of bacillus Calmette‐Guérin vaccine (BCG) to BCG+OPV administered to a cohort of children in Guinea‐Bissau during the first month of life. Within the 12 months of follow up, 73 of 3,494 (2%) of children in the BCG+OPV group and 88 of 3,467 (2.5%) of children in the BCG‐only group died, all from infectious diseases. This analysis led to the conclusion that OPV at birth protects against infant mortality. However, the effect was not significant except (1) when the results were limited to OPV administered within the first 2 days of life, or (2) for male infants only when the data were censored at the first national OPV campaign. This study is based on all‐cause mortality rates in a high infant mortality region, and no details are provided for any mechanistic or immunological effects related to OPV.

Upfill‐Brown et al. (2017) report a randomized clinical trial in Bangladesh. After vaccination with OPV at 6, 10, and 14 weeks of age, infants received either OPV (*n* = 315, which we noted appears inconsistent with the n = 307 for this arm reported in the associated methods study (Mychaleckyj et al., 2016)) or IPV (*n* = 299) at 39 weeks, with diarrheal episodes observed for the following 12 weeks. The results show fewer episodes of diarrhea for infants that received IPV than the OPV group (57% vs 63%), but reported fewer days of diarrhea per episode (6.7 vs 5.9 days) for infants with at least 1 day of diarrhea in the OPV group. Therefore, the protective effect of OPV (if any) is a ∼10% reduction (less than 1 day) in the average number of days with diarrhea, but an increased incidence of diarrhea and no information about the severity of diarrhea. The study reported a significant effect of OPV for bacterial infections in male infants, but not for viral or protozoal infections or for female infants. In addition, the study occurred in the background of significant diarrheal disease within the population, involving the majority of infants in the 12‐week follow‐up period. Despite conclusions in the study about the protective effects of OPV on infant mortality, no infant mortality data are analyzed. The Upfill‐Brown study suffers from two significant methodological issues with respect to use in health economic modeling (Upfill‐Brown et al., 2017). First, by calculating *p*‐values for diarrhea days and episodes using a zero‐truncated Poisson model, the results exclude patients with fewer than one whole day of diarrhea from comparison. This biases the conclusions towards an OPV effect. Second, infants in the study received measles and rubella vaccines during the observation window (at week 40), and as a result, any benefits from OPV are confounded by potential effects of these additional live vaccines.

In addition to the above two randomized clinical trials designed specifically to study the nonspecific immunological effects of OPV, we found a number of other studies in three general categories: (1) “natural experiments” in children receiving different levels of care (Aaby et al., 2005; Aaby et al., 2004; Andersen et al., 2018; Mogensen, Andersen, Rodrigues, Benn, & Aaby, 2017), (2) case control or observational studies in response to disease outbreaks in the general population (Chumakov et al., 1992; Shindarov et al., 1979; Voroshilova, 1989), and (3) observational studies during phase out of OPV in developed countries (Seppala et al., 2011; Sørup et al., 2016). Although a detailed systematic analysis of this literature falls beyond the scope of this manuscript, and other systematic reviews exist (Higgins et al., 2016; Kandasamy et al., 2016), when taken together, these studies do not provide a consistent or generalizable protective effect for OPV. The pediatric studies show a heterogenous set of outcomes typically characterized by some protective effect in one cohort of children but not in others (e.g., Aaby et al., 2005), or a beneficial effect for OPV given before 36 months of age but not after (e.g., Sørup et al., 2016), or a detrimental effect in boys (Benn et al., 2008) that contradicts the beneficial effect in boys reported by others (Upfill‐Brown et al., 2017). The adult studies either show observations of a correlation without evidence of causation (Shindarov et al., 1979) or speak of remarkable effects in which OPV given within hours of the onset of respiratory symptoms will effectively cure influenza or acute respiratory distress in less than a day (Chumakov et al., 1992). Finally, we note well‐established recognition that host factors affect the type and severity of immune response to antigens, with some studies demonstrating this specifically for OPV (e.g., Maldonado et al., 1997). Such host response differences must be studied and modeled before nonspecific effects of OPV can be generalized to population health.

In summary, the two controlled studies designed specifically to measure the beneficial effect of OPV (Lund et al., 2015; Upfill‐Brown et al., 2017) do not provide evidence suggestive of a measurable impact across the U.S. population. The numerous other clinical reports point to inconsistent and heterogenous results in biased or highly selective populations that defy generalization. Collectively, these clinical studies support the general concept of immunological diversity related to host factors including history of prior infections. However, the hypothesis that OPV specifically boosts immunity in such a way that would benefit human health (beyond inducing polio‐specific immunity) remains unproven. This conclusion is consistent with results of the systematic reviews of similar studies for other vaccines commissioned by the WHO. Notably, one review concluded that “the quality of the evidence, however, does not provide confidence in the nature, magnitude, or timing of nonspecific immunological effects after vaccination with BCG, diphtheria, pertussis, tetanus, or measles containing vaccines nor the clinical importance of the finding” (Kandasamy et al., 2016). We further note that the countries that have phased out OPV in the past did not report increases in infant mortality or hospital admissions, so any concerns about the phase‐out of OPV leading to potentially higher infant mortality or adverse human health effects is insufficiently supported by available evidence to date. Thus, for purposes of health economic analyses we do not see sufficient evidence of a benefit to model (i.e., no positive externalities relevant for potential inclusion in polio or COVID‐19 health economic analyses).

We further note that the recent proposal to introduce OPV in the general population suggests that a transient boost in innate immunity from OPV in the entire U.S. population could lead to disruption of the transmission of SARS‐CoV‐2 (K. Chumakov et al., 2020). While this may represent a theoretical possibility, the practical reality is quite different. With respect to SARS‐CoV‐2 transmission, the nonspecific effects (if any) of the temporary boost in innate immunity would wane, and thus leave the OPV recipients at risk of still becoming infected with SARS‐CoV‐2 as it continues to transmit through the population. Specifically, unlike a vaccine that induces SARS‐CoV‐2‐specific immunity, any OPV benefit would depend on exposure to SARS‐CoV‐2 occurring after OPV‐induced innate immunity, which would only occur in some people. The receipt of OPV could lead to simultaneous OPV infection in individuals already infected with SARS‐CoV‐2, which could affect the presentation of either or both diseases in unpredictable ways.

### 3.5 Logistics and Risk–Risk Tradeoffs

The logistics of administering a vaccine, even an oral one, to all (or most) Americans within a short period of time will likely prove intractable due to the lack of existing mechanisms to get the doses to individuals, which we should recognize as an important aspect of roll out of any future SARS‐CoV‐2 vaccines. In addition, the risks of bringing people in contact to administer the OPV doses, which come in multidose vials, would imply risks of potential transmission of SARS‐CoV‐2 at the OPV administration sites.

Consistent with the global commitment to contain live polioviruses (World Health Assembly, 2018) and meetings with the poliovirus vaccine manufacturers (World Health Organization and UNICEF, 2018), the U.S. created its National Authority for Containment (NAC) (Centers for Disease Control and Prevention, 2020). The U.S. NAC “strongly encourages facilities to destroy all poliovirus containing samples that are not deemed essential” and this includes OPV‐related viruses.

## 4 DISCUSSION

This analysis suggests that health and financial resources of reintroducing OPV into the U.S. population could lead to substantial health and financial costs with no expected identifiable benefits. U.S. public health officials should consider the risks and costs of using OPV in the U.S. and recognize the opportunity costs of using the estimated over $4.4 billion for nonspecific benefits of OPV instead of for developing specific vaccines and delivery strategies for SARS‐CoV‐2 vaccines.

Any decision to restart OPV use in the U.S. should consider the logistics and realistic estimates of expected costs and benefits, and we suggest that these should go through review by the ACIP. Reintroducing OPV into the U.S. population with substantially less than 100% coverage could lead to unexpected and asymptomatic transmission of OPV‐related viruses due to the considerable number of young Americans who lack any prior exposure to live polioviruses (Thompson et al., 2015; Thompson et al., 2012). Due to current levels of vaccine confidence (National Vaccine Advisory Committee, 2015), not all Americans would likely even accept a 100% effective COVID‐19 specific vaccine. We should expect even lower compliance with OPV because it would not provide any expected effective or durable immunity for SARS‐CoV‐2. Any introduction of OPV in the U.S. without proper isolation control, even in the context of small numbers of doses potentially given preferentially to high‐risk individuals for COVID‐19 as part of a clinical trial, would run the risk of restarting chains of OPV transmission. Such chains of transmission, which like in Israel would occur asymptomatically, could lead to the transmission of VDPVs at a level that could lead to a perceived or actual need for broader OPV use. We see no justification for incurring any risks or costs associated with introducing OPV to obtain transient, nonspecific effects (if any) against SARS‐CoV‐2.

The situation in the U.S. differs substantially from that in most countries, which currently use licensed bOPV in their national immunization programs. Due to the conditions in these countries (e.g., higher risks of importations, relatively low vaccine coverage rates, crowding, hygiene, etc.), IPV does not stop or prevent the transmission of live polioviruses (wild or OPV‐related). Thus, in most countries, the national immunization programs must use OPV to stop and prevent the transmission of wild polioviruses and cVDPVs (Thompson et al., 2015). The GPEI strategy requires that for each of the three poliovirus serotypes, OPV‐using countries must achieve and maintain high coverage with OPV to end all WPV transmission contemporaneously everywhere, after which successful global eradication of indigenous transmission of the WPV serotype can be certified (as occurred for serotype 2 in 2015 and serotype 3 in 2019 (World Health Organization, 2015, 2019)). After certification, the GPEI plan includes globally coordinated cessation of the use of the OPV serotype to end all VDPVs (i.e., except for any emergency OPV use), as occurred for serotype 2 in 2016 (Farrell et al, 2017).

The reason that global polio eradication continues to take so long is because vaccinating nearly all (or at least enough) susceptible individuals everywhere in the world is difficult. New children born every day necessitate ongoing efforts to reach new birth cohorts, and countries also need to catch up the individuals missed in the past. In OPV‐using countries, OPV does some of the work with respect to catching up missed children due to its secondary spread, but as noted, that secondary spread comes with some risks. Nonetheless, in countries that use OPV, any benefits of nonspecific boosting of immunity from OPV use would occur secondarily to the benefits of achieving and maintaining high OPV coverage, which is required for polio eradication. For these countries, if the argument of nonspecific effects of OPV use with respect to preventing some COVID‐19 provides a means to increase polio vaccine coverage, then any benefits would seem aligned with existing national policies and strategies.

The GPEI continues to pursue its mission to permanently end all transmission of wild polioviruses and stop all cases of poliomyelitis, and any recommendations related to the use of OPV should occur in the context of full awareness of the current global situation. The situation with continued serotype 2 transmission in Africa at a time that transmission should have ended according to the global strategy (Kalkowska et al., 2020) suggests the need to develop better strategies and/or better OPV vaccines that do not come with the same VAPP and VDPV risks (if possible) (Thompson, 2019). Such efforts to develop new OPV strains (Van Damme et al., 2019) are underway, and clinical trials for these will likely reveal the extent to which they reduce or eliminate the risks of OPV as well as the extent to which they sustain the benefits.

Polio eradication could still represent one of the greatest success stories of public health cooperation on a global scale, but only if national and global health leaders focus on achieving high performance of cost‐effective strategies that allow achievement of the goal. National health policy leaders face many choices about which poliovirus vaccine(s) to use in their national immunization programs, and how to best administer and finance them (Thompson & Kalkowska, 2020a).

Recognizing the logistical challenges of delivering any vaccine to the entire U.S. population to respond to a pandemic, we expect that public investments in strategies based on nonspecific effects could lead to unintended consequences. For example, if OPV is the first vaccine given to the American population for SARS‐CoV‐2, then this may impact the willingness to accept vaccines that induce specific immunity for SARS‐CoV‐2. We suggest that any efforts to induce nonspecific innate immunity in the U.S. should start with existing licensed live vaccines (e.g., MMR) (Fidel & Noverr, 2020) instead of vaccines that the current standards of care in the U.S. already exclude (e.g., OPV, BCG). All vaccines used in the U.S. should also continue to undergo review by the ACIP, because ACIP recommendations play a critical role in public financing and ensuring the compensation of any injuries associated with recommended vaccines.
